# Commentary: Application of personal computers in the laboratory

**DOI:** 10.1155/S1463924681000473

**Published:** 1981

**Authors:** R. W. Arndt


**An important announcement for subscribers to**

THE JOURNAL OF AUTOMATIC CHEMISTRY

With effect from Volume 4 (1982) this journal will be published by Taylor & Francis Ltd.

All records are being transferred from United Trade Press Ltd to Taylor & Francis Ltd, so the continuity of your subscription will be assured. When your current subscription expires, you will automatically receive a renewal notice from Taylor & Francis Ltd.

Since 1798, Taylor & Francis Ltd have been publishing scientific and technical journals, and have established a tradition of expertise and service which will be brought to bear on this important addition to their list of journals.

If you have any queries regarding the change of publisher, or you would like to receive further information about Taylor & Francis Ltd and their publishing activities, please contact Mr Keith Courtney, Marketing Manager.

Taylor & Francis Ltd

4 John Street, London WC1N 2ET

Tel: 01-405 2237 Telex: 858540

### Commentary

### Application of personal computers in the laboratory

The explosion in the personal computer market has now reached the stage where they are conspicuous in our daily lives. Sales are growing at a rate of 30-50% per annum and this year they can be expected to surpass sales of analytical instrumentation (personal computer sales are worth $1500 million compared with an analytical instrumentation market of $1300 million). Cheaper units with improved specifications keep appearing, reliability has improved and there are now systems with colour graphics available. The price of a personal computer is so low that practically everybody can afford one. The multitude of systems is bewildering and makes proper selection look like an insurmountable task.

As personal computers affect more and more facets of our lives, they are also sweeping into the analytical laboratory, leaving their mark on both operation and organisation. However, the reception they are being given in the laboratory so far has been decidedly cool. The forerunner of the personal computer in the laboratory, the HP-85, has not really "paved the way" and although computers *per se* are now of fundamental importance in a modern laboratory, analytical chemists still carefully evaluate any changes they may cause in order not to endanger continuity. Nevertheless, before long, personal computers will invade the sanctity of the laboratory in a big way and many reports of user experience are already appearing in the literature. The development of computerisation is repeating itself; decentralisation, this time at the level of the microcomputer and local computing power, with networking at a later stage for collating and archiving purposes. A sea of possibilities is opening and the task is to sail the proper course through it.

At this stage, the editors of the *Journal of Automatic Chemistry* would like to remind the readers of the purpose of the journal. We were dismayed to see there was no forum for the exchange of information and experience gained in automating schemes introduced at individual laboratories. The journal was introduced to provide this forum and I would like to appeal to the readership to make full us of it during this exciting development with personal computers. Report on your application, and quickly!

Introducing personal computers to the laboratory is not easy, raising many questions to which there are no firm answers. Which make should be used? How big should the memory be? Are floppy-disk units needed for back-up storage? Is the IEEE-488 bus preferable, or should the user go for the RS232 for interfacing? Are interrupt systems advisable with personal computers? What software is available? · And what about service and maintenance? Does an installation bring the expected return? Questions abound and your answers should be reported.

*R.W. Arndt*

